# Food abundance in men before puberty predicts a range of cancers in grandsons

**DOI:** 10.1038/s41467-022-35217-1

**Published:** 2022-12-06

**Authors:** Denny Vågerö, Agneta Cederström, Gerard J. van den Berg

**Affiliations:** 1 Centre for Health Equity Studies, CHESS, Department of Public Health Sciences, Stockholm University, Stockholm, Sweden; 2 University of Groningen, University Medical Center Groningen, Groningen, the Netherlands

**Keywords:** Risk factors, Cancer epidemiology, Epigenetics

## Abstract

Nutritional conditions early in human life may influence phenotypic characteristics in later generations. A male-line transgenerational pathway, triggered by the early environment, has been postulated with support from animal and a small number of human studies. Here we analyse individuals born in Uppsala Sweden 1915–29 with linked data from their children and parents, which enables us to explore the hypothesis that pre-pubertal food abundance may trigger a transgenerational effect on cancer events. We used cancer registry and cause-of-death data to analyse 3422 cancer events in grandchildren (G2) by grandparental (G0) food access. We show that variation in harvests and food access in G0 predicts cancer occurrence in G2 in a specific way: abundance among paternal grandfathers, but not any other grandparent, predicts cancer occurrence in grandsons but not in granddaughters. This male-line response is observed for several groups of cancers, suggesting a general susceptibility, possibly acquired in early embryonic development. We observed no transgenerational influence in the middle generation.

## Abstract (web summary)

Nutritional conditions experienced early in life may influence the disease risk of future children and grandchildren. Here the authors report that food abundance among boys before puberty associates with the relative risk of a range of cancers in grandsons, but not in granddaughters.

## Introduction

Animal studies have demonstrated that environmental conditions early in life can change phenotypic characteristics in subsequent generations through epigenetic pathways, such as methylation, histone modification and noncoding RNAs^1–3^. In particular, nutritional conditions early in life may trigger epigenetic changes that are heritable and may affect health and disease outcomes in more than one generation of offspring. Classic examples of gene silencing, for instance X-inactivation, parental imprinting and metastable epialleles, were first discovered in mice.

Studies on the Agouti mouse provide examples of epigenetic transgenerational inheritance; food supplements have been shown to change phenotypic characteristics in five successive generations^4–7^. Offspring of mice fathers who suffered periconceptional food deprivation experienced a change in growth-and metabolic-related parameters, in particular a decrease in serum glucose^8^. Feeding young male mice with high-fat diet-induced obesity, which was recapitulated in their offspring and grand-offspring^9^. Chen et al.^10^ found that paternal exposure to a high-fat diet was associated with metabolic dysfunction in offspring, probably mediated by sperm tRNAs.

This line of research has led to a new interest in the contribution of paternal experience prior to conception to offspring health and disease^11,12^. The development of germline stem cells into mature sperm and oocytes is tightly regulated. Epigenetic manifestations in germ cells may together carry a molecular memory of prior environmental exposures. A growing literature discusses how molecular memories of the past could be carried forward to the next generation and affect offspring development in mammals^3,13–15^. Jazwiec et al.^15^ suggested that paternal obesity might influence placental vessel structure and Pepin et al.^16^ observed an association between the sperm epigenome, placental development and offspring metabolism.

Recent research has shown that the sperm epigenome is highly responsive to environmental influences^16–19^. Notably, the content and mobility of human sperm respond rapidly to changes in diet^20,21^. Experimenting with a diet rich in sugar, Nätt et al.^21^ found that transfer-RNA-derived small RNAs (tsRNA) in human sperm were upregulated after just a week of exposure. tsRNAs increase sharply in number during late spermatogenesis and epididymal maturation. As the maturing sperm travels though the epididymis it is able to pick up nutritionally based molecular signals^22,23^. It has been suggested that the latter pathway is instrumental in carrying information from soma to germ cells^10,24,25^, breaking the so-called Weismann barrier^26^. A diet rich in methyl donors could contribute to methylation of sperm DNA (which is reset, but not fully, after fertilisation) or affect microRNAs in semen fluid, with potential consequences for offspring gene expression and development^6,27^.

Pembrey et al.^28^ discuss lessons from animal studies (especially sex-specific transgenerational response) for the study of humans. They conclude that life course studies of human cohorts should be reframed to include exposures in previous generations of men and women. In humans, it is well known that maternal nutrition and placental function influence foetal growth. It is also well established that the foetal environment can influence offspring phenotype through biological programming in utero, established by epigenetic regulation of foetal gene expression.

In studies that relate nutritional exposure to later-life outcomes, foetal nutrition has been linked both to circulatory disease^29,30^ and to cancer^31–33^ Frankel et al.^34^ used detailed information collected from 1937 to 1939 about food intake among British children. They demonstrated that an energy-rich diet in childhood was linked to cancer risk in later life (most clearly in cancers not related to tobacco smoking) among both men and women if family social circumstances were controlled for. Vågerö et al.^35^ found that food abundance among men in the period before puberty predicted cancer death in their grandsons, but not their granddaughters.

Effects of food deprivation early in life on adult health were reviewed by ref. 36. Inter- and transgenerational consequences of severe food deprivation among humans have been addressed by studying historical famines, such as the Dutch Hunger Winter 1944–45^37^, the Ukrainian famine 1932/33^38^ and the Chinese famine of 1959–61^39^. Starvation among pregnant women was linked to a number of outcomes in their offspring, such as demethylation of the imprinted *IGF2* gene^37^, type 2 diabetes^38^ and schizophrenia^39^. Veenendaal et al.^40^ found that men, but not women, who were starved in utero tended to have obese offspring.

From historical cohort studies, there is a suggestion of a specific male-line inter- or transgenerational effect from early nutrition. Food abundance or food shortage in boys/young men may trigger an elevated cancer, circulatory, diabetes or all-cause mortality in children or grandchildren^35,41–44^. Four of these five studies exploit data on harvest conditions as a source of contextual variation in nutritional conditions. Historically, good harvests usually mean higher consumption of food such as cereals and vegetables, both of which are methyl donors.

In the current paper, we further explore the hypothesis of a male-line transgenerational pathway to cancer, by examining cancer occurrence in men and women in the Uppsala Multigeneration Study database by their paternal and maternal grandparents’ access to food before puberty. Cancer occurrence refers to a primary cancer tumour as recorded in the Swedish Cancer Registry or a death from cancer registered as an underlying cause in the Swedish Cause-of-Death Registry. Pre-mortality cancer diagnoses are observed earlier in life than causes of death, making analyses of such data less sensitive to assumptions about censoring due to other death causes. We also explore cancer events in the middle (G1) generation in a similar manner to clarify whether any transgenerational influence is also manifest there.

We find that access to abundant food in young men (G0) predicts a range of cancers in their grandsons (G2) via a paternal pathway. Any influence on their granddaughters is much more limited. Food access of other grandparents was not associated with G2 cancer occurrence. The broad spectrum of response in G2 men is compatible with a susceptibility to cancer that is acquired in early embryonic development. We see little evidence of this transgenerational influence in the middle (G1) generation.

## Results

We were able to link three generations, denoted as Generations 0, 1 and 2 (G0, G1 and G2), to each other. From regional harvest statistics, available for the years 1874‒1910, we collected information about harvest yields for the region where the grandparental generation (G0) grew up. G0 pre-pubertal food access was classified as good/abundant, poor/very poor or intermediate. Calculated across all calendar years and regions, the proportion of G2 whose G0 grandparents were exposed to good/abundant harvests varied between 4.7% and 4.9% depending on which grandparent we refer to. The corresponding proportion of G1 whose G0 mother or father was exposed to good/abundant harvest varied from 4.6% to 4.8%. They were compared to the group with intermediate ancestral exposure, the size of which varied between 89.5% and 91.4% (Supplementary Table 1).

Cancer occurrence was followed up from 1 January 1961 to 31 December 2017. The total observed number of cancer events in G2 was 2355 for those with both paternal or both maternal grandparents growing up rurally. Among G2 with urban or mixed urban/rural grandparental background we observed 1067 cancer events. For G1 with both G0 parents growing up rurally there were 1599 cancer events (Table 1).

**Table 1:** Number of persons, person-years at risk and cancer events in G2 and G1 by G0 rural/urban background in family lines where G0 is born 1865‒1900 with full covariate data

*Ancestry*	*N*	Person years 1961–2017	Cancer events 1961–2017
*G2 men*			
Paternal G0s are rural	2240^a^	124256	466
Maternal G0s are rural	2070^a^	117593	587
Paternal G0s are urban or mixed	989	55713	222
Maternal G0s are urban or mixed	926	52173	225
*G2 women*			
Paternal G0s are rural	2098^b^	120559	641
Maternal G0s are rural	2023^b^	117518	661
Paternal G0s are urban or mixed	955	55602	304
Maternal G0s are urban or mixed	884	51654	316
*G1 men*			
G0 parents are rural	1960	74146	839
G0 parents are urban or mixed	1817	63940	709
*G1 women*			
G0 parents are rural	1821	73445	760
G0 parents are urban or mixed	1605	60958	651

^a^For 175 of these men there are full data on both the paternal and the maternal side.

^b^For 202 of these women there are full data on both the paternal and the maternal side.

### Total cancer occurrence in G2 men and women

Initially, we analysed total cancer occurrence in G2 separately for those of whom both paternal and/or both maternal grandparents grew up in the countryside (Table 2) and for those with an urban or mixed grandparental background (Supplementary Table 2), as rural G0s were more dependent on regional harvest yields than were G0 urban dwellers (Supplementary Appendix). Urban dwellers were those growing up in any of the ten largest cities in Sweden, seven of which were sea ports.

**Table 2:** Cancer occurrence among G2 men and women by G0 food access, restricted to G0s with rural childhoods: hazard ratios (HR)^a^ and 95% confidence interval (95% CI) (Cox proportional hazard models) for total cancer, using intermediate food access as reference group

	*G2 men*			*G2 women*		
*Food access of*	Hazard Ratio	95% CI	*p* value	Hazard Ratio	95% CI	*p* value
Paternal grandmother						
Good/abundant	1.08	0.54–2.12	0.808	0.97	0.64–1.47	0.875
Poor/very poor	1.33	0.82–2.14	0.243	0.95	0.64–1.43	0.816
Paternal grandfather						
Good/abundant	**3.05**^**b**^	**1.98–4.69**	**4.1E-07**	1.14	0.82–1.59	0.443
Poor/very poor	0.82	0.49–1.39	0.813	1.22	0.85–1.75	0.281
*Person years*	124,256			120,559		
*Cancer events*	466			641		
Maternal grandmother						
Good/abundant	0.89	0.58–1.37	0.596	0.9	0.69–1.38	0.886
Poor/very poor	1.20	0.84–1.70	0.322	0.77	0.50–1.20	0.248
Maternal grandfather						
Good/ abundant	1.14	0.74–1.76	0.551	1.23	0.83–1.83	0.305
Poor/ very poor	1.04	0.69–1.57	0.840	0.99	0.65–1.51	0.967
*Person years*	117 593			117 518		
*Cancer events*	587			661		

Bold figures: Estimated HR significantly higher than 1, two-tailed test.

^a^Controlling for social and demographic factors, plus G0 partner’s and G1 parent food access

^b^Gender interaction: *p* = 0.00002

Table 2 shows the hazard ratios (HR) for a cancer event 1961‒2017 in G2 men and women by their maternal or paternal grandparents’ food access during ages 9–12 (boys) or 8–10 (girls), typically just before puberty. Access to good/abundant food in paternal grandfathers (PGF) who grew up in the countryside was linked to total cancer occurrence in their G2 grandsons (HR = 3.05; 95%CL = 1.98–4.69), but not their granddaughters (HR = 1.14; 95% CL = 0.82–1.59). This represents a significant interaction between G0 food access and G2 sex (*p* = 1.76E-05). Total cancer was not linked to the food access of maternal grandfathers or paternal or maternal grandmothers (Table 2).

The hypothesis of a specific pathway from G0 (paternal grandfather’s) food abundance to cancer occurrence in G2 (grandsons) gains support (*p* = 4.1E-07). This was also our a priori hypothesis, based on a previous study^35^ as well as literature cited in the ‘Introduction’^42^.

Grandchildren of urban or mixed G0s were treated as one group. For these, we saw no association between regional harvest yields and G2 cancer occurrence (Supplementary Table 2). Analyses below are therefore based on those G2 and G1 both of whose paternal and/or maternal G0 ancestors grew up in the countryside.

### Cancer occurrence in G2 for six groups of cancer

We further explored the finding of a pathway from paternal grandfathers to their grandchildren by looking at broad groups of cancer. We reasoned that a high number of cancer events as a response to ancestral food abundance could either be caused by a general susceptibility to cancer or be driven by a specific group of cancers. Knowledge and classification of cancers have changed during the long follow-up period, from 1961 to 2017. However, the Swedish Cancer Registry allows classification of all cancers for the whole period according to the 7th International Classification of Disease (ICD7)^45,46^. Thus, we were able to analyse the whole period by six broad ICD groups of cancer, using ICD7 (Table 3).

**Table 3:** Cancer occurrence in G2 men and G2 women by paternal grandfathers’ (G0) food access in childhood, restricted to G0s with rural childhoods: hazard ratios (HR)^a^ and 95% confidence intervals (95% CI) (Cox proportional hazard models) for six ICD 7 classes of cancer using intermediate food access as reference group

*Food access of*	*G2 men* person years = 124,256		*G2 women* person years= 120,559	
Paternal grandfathers	Hazard Ratio (95% CI)	*p* value	Hazard Ratio (95 CI)	*p* value
	*Cancer in buccal cavity and pharynx (140–148)*			
	10 events		3 events	
Good/abundant	**27.50 (2.28–332.0)**	**0.009**	**24.3 (5.38–110)**	**0.00003 B+**
Poor/very poor	0 (0.0-		0 (0.0-	
	*Cancer in digestive organs and peritoneum* (150–159)			
	105 events		60 events	
Good/abundant	**4.28 (1.82–10.1)**^**b**^	**0.0009 B**+	0.69 (0.16–3.01)^b^	0.624
Poor/very poor	0.75 (0.176–3.17)	0.693	1.24 (0.29–5.29)	0.774
	*Cancer in respiratory system (160–165)*			
	40 events		49 events	
Good/abundant	2.86 (0.72–11.28)	0.134	0.72 (0.10–5.38)	0.750
Poor/very poor	0.43 (0.06–3.23)	0.415	0.98 (0.20–4.80)	0.982
	*Cancer in breast and genito-urinary organs (170–181)*			
	171 events		379 events	
Good/ abundant	**2.02 (1.14–3.58)**	**0.016**	0.98 (0.64–1.52)	0.940
Poor/ very poor	0.74 (0.29–1.90)	0.532	0.98 (0.60–1.61)	0.942
	*Cancer in other and unspecific sites (190–199)*			
	92 events		116 events	
Good/abundant	**3.19 (1.61**–**6.33)**	**0.0009 B**+	1.66 (0.83–3.33)	0.151
Poor/very poor	1.63 (0.72–3.70)	0.242	**1.94 (1.02–3.67)**	**0.043**
	*Cancer in lymphatic and hematopoietic tissues (200–209)*			
	37 events		25 events	
Good/abundant	**4.73 (1.32–16.97)**	**0.017**	0.54 (0.06–4.80)	0.581
Poor/very poor	0.56 (0.07–4.25)	0.577	1.45 (0.41–5.12)	0.560

Bold figures: HR estimate significantly higher than 1, two-tailed test.

**B+** survives a Bonferroni test (*p* < 0.002)

^a^Controlling for social and demographic factors, plus G0 partner and G1 parent food access

^b^*p* for interaction G0 harvest/G2 gender = 0.006

Looking first at the general pattern, we noticed that good/abundant access to food in G0 men (PGF) was associated with elevated hazard ratios (HR > 1) in their G2 grandsons for all six cancer groups; a multiple response which is unlikely (*p* = 0.016) to be a chance occurrence. In fact, all six HRs were larger than 2. The hypothesis of a general susceptibility to cancer must be considered.

When we examined each one of the six broad cancer groups in isolation, we observed that, in five of the six groups, HRs among ancestrally exposed G2 men were significantly raised, using traditional criteria (*p* < 0.05). For instance, the HR for *cancer of the digestive organs and peritoneum* is 4.28 (95% CI 1.82–10.1); the HR for *other and unspecified cancers* is 3.19 (95% CI 1.61–6.33) and for *lymphatic and hematopoietic cancers* it is 4.73 (95% CI 1.32–16.97). In contrast, HRs among ancestrally exposed G2 women in these three cancer groups are all compatible with the null hypothesis.

We had no a priori hypothesis about which cancer group, if any, should be particularly affected. A cautious approach would therefore be to apply a Bonferroni correction^47^ for multiple testing (tests in G2 men and women, by good and poor G0 food access, for six groups of cancer= 24 tests), using a *p* value <0.002 (0.05/24) (see method section).

For *Cancer of the digestive organs and peritoneum (ICD7 150*–*159*) and for *Other and unspecific cancers (ICD7 190–199)* differences are both highly statistically significant (both *p* ≤ 0.0009) even after a Bonferroni correction. Tests for interaction suggest that the response to G0 food access is modified by G2 gender (*p* = 0.006) for cancer of digestive organs and peritoneum.

However, even if we combine these two broad groups of cancer they do not explain why G2 men with ancestral exposure (PGF) to abundant food have a threefold excess of total cancer. The remaining four broad groups contribute to this result. Their combined hazard ratio equals 2.6 (95% CI 1.5–4.3, *p* = 0.00026).

Cancer occurrence in the buccal cavity and pharynx is strikingly high in both men and women if paternal grandfathers experienced good/abundant food, but the very small number of cancer events (men *n* = 10; women *n* = 3) calls for caution.

### Cancer occurrence in G1 by G0 food access

While our hypothesis concerned a transgenerational response, therefore focusing on the association G0 > G2, we also examined whether this paternal pathway manifested itself in G1 men. Table 4 suggests that this is not the case. For G1 men who were sons of G0 fathers with good/abundant pre-pubertal access to food the hazard ratio (all cancer) was 0.81 (95% CI 0.6–1.1). The corresponding HR for G1 women was 0.90 (95% CI 0.7–1.3).

**Table 4:** Cancer occurrence in G1 men and women by G0 fathers’ food access in childhood

*Food access of*	*G1 men* person years = 74,146		*G1 women* person years = 73,445	
G0 father	Hazard Ratio (95% CI)	*p* value	Hazard Ratio (95 % CI)	*p* value
	*All Cancer (140–209)*			
	*839 events*		*760 events*	
Good/abundant	0.81 (0.59–1.12)	0.208	0.90 (0.65–1.25)	0.553
Poor/very poor	1.08 (0.78–1.50)	0.650	0.71 (0.46–1.09)	0.114
	*Cancer in buccal cavity and pharynx (140–148)*			
	*11 Events*		*8 events*	
Good/abundant	0 (0-∞)	-	1.92 (0.20–18.82)	0.574
Poor/very poor	4.94 (0.95–25.77)	0.058	3.93 (0.44–34.81)	0.219
	*Cancer in digestive organs and peritoneum* (150–159)			
	182 events		149 events	
Good/abundant	0.45 (0.18–1.11)	0.085	0.58 (0.24–1.45)	0.244
Poor/very poor	0.90 (0.41–1.93)	0.778	0.60 (0.22–1.63)	0.314
	*Cancer in respiratory system (160–165)*			
	100 events		38 events	
Good/abundant	1.47 (0.69–3.13)	0.314	0.85 (0.20–3.67)	0.825
Poor/very poor	0.26 (0.03–1.83)	0.174	0.59 (0.08–4.38)	0.608
	*Cancer in breast and genito-urinary organs (170–181)*			
	318 events		335 events	
Good/abundant	1.*05* (0.65–1.70)	0.839	0.84 (0.51–139)	0.497
Poor/very poor	1.09 (0.63–1.88)	0.755	0.83 (0.46–1.53)	0.558
	*Cancer in other and unspecific sites (190–199)*			
	149 events		170 events	
Good/abundant	0.68 (0.29–1.56)	0.359	0.94 (0.47–1.88)	0.868
Poor/very poor	1.26 (0.60–2.60)	0.539	0.43 (0.14–1.34)	0.146
	*Cancer in lymphatic and hematopoietic tissues (200–209)*			
	76 events		55 events	
Good/abundant	0.36 (0.09–1.50)	0.162	1.76 (0.67–4.64)	0.255
Poor/very poor	1.62 (0.69–3.79)	0.269	0.97 (0.23–4.04)	0.968

^*^HRs after controlling for social and demographic factors, plus G0 partner’s food access. No Bonferroni correction.

Restricted to G0s with rural childhoods: hazard ratios (HR)* and 95% confidence interval (95% CI) (Cox proportional hazard models) for total cancer and six ICD7 groups of cancer using intermediate food access as reference group

For G1 men and G1 women, results for the six broad groups of cancer are all compatible with the null hypothesis of no paternal (G0) influence on G1 (Table 4).

It should be noted that the comparison between G1 and G2 was restricted by the design of the data collection. Specifically, generation 1 was followed up at a later stage in their life than generation 2. In the analysed sample the median age of cancer onset was 60 in G2 men and 51 in G2 women compared to 73 and 71 for G1 men and women (Supplementary Table 3). As a sensitivity test we restricted the follow-up period for G1 to the period 1961–1988. Mean age-at-risk for G1 during this restricted period was comparable to the mean age-at-risk in G2 analyses. This restriction did not change results for G1 men in any way (Supplementary Table 4).

## Discussion

We observed rather robust associations between food abundance in the period before young men’s puberty and cancer occurrence in their grandsons. We considered 1) bias, 2) confounding from social factors, and 3) chance as explanations for these associations. If they were indeed likely to be causal, we considered alternative pathways for this influence.

Firstly, we considered bias*:* Harvest yields vary year by year and region by region. Thus, the availability of food in a specific year and a specific region can be seen, in part, as the result of an *experiment by nature*. Using natural experiments in health studies avoids bias and reduces confounding^48^. The argument is that the random element of G0 exposure (variation in harvest yields) is most likely to be related to later health through its association with G0 food intake. Using average harvest yields as individual exposure does mean some misclassification of individual exposure. However, this measure of food access is not influenced by any knowledge of future cancer outcomes -there is no selection bias. Such misclassification of individual food access is therefore non-systematic and should not bias estimates of cancer hazards.

Secondly, we considered social confounding: Social continuity in advantage/disadvantage across generations, partly driven by family influences on children’s and grandchildren’s education and health, is a well-known phenomenon in the sociological literature^49,50^. The early social and family environment does indeed predict health in successive generations in the Uppsala Multigeneration Study database^49,51^.

Lawlor et al.^52^ analysed the importance of the early social environment for adult cancer in the Swedish population and found that total cancer mortality was higher among those growing up in working-class families. Stomach cancer, for instance, was directly linked to early family factors. G2 growing up in working-class families (G1 households in our study) mostly lived in urban areas but would have been recruited from rural labouring classes. Vorha et al.^53^, reviewing cancer mortality studies, concluded that childhood disadvantage was linked to gastrointestinal cancer risk in adults.

We would expect young men (G0) from families in disadvantaged social circumstances to have had access to a smaller-than-average share of the annual harvest, regardless of whether it was a good or bad harvest. Thus, any social confounding may obscure the associations we did observe between G0 food abundance and G2 cancer occurrence. We controlled for a number of social and demographic circumstances, including G0 birth year, G1 education, social class and income (for a full list see method section). This tended to increase hazard ratios for a cancer event (total cancer) very slightly (from 3.01 to 3.05), suggesting that social confounding does not explain the observed association between ancestral food abundance and cancer in G2 men. It should also be noted that one would expect social confounding to give similar results in G1 and G2, contrary to our findings.

Wylde et al.^54^ found, in animal experiments, that paternal age at conception affects offspring longevity over two generations, along patrilines as well as matrilines. They also demonstrated that this pathway is independent of the diet and stress exposure of G0. Carslake et al.^55^ used Swedish data on 3.6 million offspring to study human phenotypes as a function of paternal age and found that the associations were very small if the analysis controls for maternal age. However, it is known that a high paternal age is linked both to epigenetic changes and mutations in sperm^56^. To proceed, we performed a sensitivity test by introducing G0 men’s age at the birth of G1 in analyses of G2 cancer occurrence. Supplementary Table 5 shows that the estimated hazard ratio for grandsons of paternal grandfathers with access to good/abundant food, hardly changed at all, i.e. from 3.05 to 3.04. Thus, G0 paternal age does not confound our result.

In a Supplementary Appendix to the paper, we provide evidence about the historical context in which G0 grew up and argue that a good harvest causes higher transitory income in households when G0 were children. This is more likely to influence G0 food habits in childhood than smoking and drinking in adolescence and adulthood. We acknowledge that an increase in physical activity among children, for instance by helping with an abundant harvest, is possible. The data do not allow us to study the extent to which this took place.

Behavioural pathways in grandparents’ influence on grandchildren’s cancer risk were studied in a systematic review^57^. They concluded that grandparents often had an adverse impact on risk factors such as ‘weight, diet, physical activity and tobacco’. However, it seems very unlikely that any such influence from well-fed paternal grandfathers should be more negative than that of any other grandparent. A reasonable conclusion is that grandparents´ cultural influence on their grandchildren’s behaviour does not confound our results.

Thirdly, we considered the role of chance*:* In line with our à priori hypothesis the hazard ratio for all cancer was elevated among grandsons of paternal grandfathers exposed to food abundance, but not in any other combination of grandparents (G0), grandchildren (G2) and food access (good/abundant or poor/very poor). The likelihood of finding a threefold excess by chance is very small. Elevated cancer hazards (HR > 1) were estimated for all six broad groups of cancer in men, an unlikely result if the null hypothesis of no general risk is true.

This observed pattern of cancer occurrence is therefore compatible with a general cancer susceptibility among men, defined as a pathogenic response across a range of cancer localisations, triggered by ancestral food abundance. We considered the alternative hypothesis that the pattern is driven by a specific group of cancers. We found no support for that alternative hypothesis.

If ancestral food experience does indeed predict a range of cancer outcomes, what are the pathways? Studies in mice and rats have demonstrated that paternal and maternal diet may induce phenotypical change in offspring via molecular alterations in germ cells^1^. Proof-of-principle for the hypothesis of a male-line transgenerational response in humans, triggered by food abundance in pre-puberty, was first claimed in a 2006 paper by ref. 42. The pathway was hypothesised to be epigenetic. Their findings were based on mortality data and on data on prepubertal smoking and offspring BMI, rather than on epigenetic data. They speculated that a male-line transgenerational effect could be mediated (at least in part) by something carried on the Y chromosome. Epigenetic inheritance involves cross talk between sex chromosomes and autosomes and parent-of-origin transmission occurs across the entire genome^6,28^.

X and Y chromosomes exchange genetic material in the so-called pseudo-autosomal pairing regions during meiosis and this material may carry sequences for multiple cancers. In women, one of the two X chromosomes is inactivated, but a number of tumour suppressor genes, located in the non-pseudoautosomal region (‘EXITS genes’), escape inactivation, allowing biallelic expression^58^. Thus, the two X chromosomes in women provide extra protection against cancer. The tumour suppressor gene *P53* on the short arm of chromosome 17 (the so called guardian of the genome^59^) interacts with a network of genes on the X, contributing to a widespread male bias in cancer occurrence^60^.

Our support for the hypothesis of a male-line transgenerational response in humans to early nutrition is based on data of cancer occurrence over a 57-year period, 1961‒2017. Cancer occurrence in general grew slowly but steadily in Sweden during this period^61^. If it is indeed possible that nutritional signals, picked up in pre-puberty, are carried forward across generations to cause disease, cancer would seem to be a likely candidate. A general cancer response in G2 men points to events in their early embryonal/foetal development, even before differentiation into endoderm, ectoderm and mesoderm cell lines.

In boys, onset of puberty and development of the testis is linked to the transformation of spermatogonial stem cells into mature sperm. The process of spermatogenesis is accompanied by dramatic changes in gene expression^62^. Numerous small noncoding RNAs are exclusively or preferentially expressed in testis or germ cells in humans and mice. In men, Sertoli cells, which are part of the seminiferous tubules of the human testis, regulate nutrients and growth factors which support developing germ cells. Animal studies suggest that expression of the *p53* tumour suppressor gene in Sertoli cells may be downregulated during the peri-pubertal period^63^. It has been suggested that the period immediately before puberty is a critical or sensitive window for germ line programming^12,42,64,65^. A rich food intake before puberty may also influence the timing of puberty by triggering its early onset^66^.

As early as 1904 Andrea Rabagliati, a Scottish surgeon concerned with the increasing proportion of deaths due to cancer, suggested that overfeeding is the predisposing cause of cancer^67^. Frankel et al.^34^ were more specific and concluded that higher levels of energy intake in childhood increase the risk of later development of cancer. They did not speculate about whether energy intake could have effects spanning more than one generation.

Abundant harvests also mean that larger quantities of food are stored. Aflatoxin is a well-known mutagen of the *P53* tumour suppressor gene^68^, related to mould in stored food such as cereals or vegetables. Rye, a common cereal in Sweden in 1874‒1910, hosts particularly large concentrations^69^. Aflatoxin is also linked to epigenetic changes^70^. Could aflatoxin have caused de novo mutation in a tumour suppressor gene in G0 men? The lack of cancer response in G1 suggests that such a mutation is not an explanation.

An epigenetic change in the G0 germ line in the period before puberty, triggered by excess energy intake or by carcinogens in food, could be expected to affect G1. However, epigenetic changes carried forward to G1 could be reversed in the pre-implantation embryo and reintroduced later. Epimutation in G1 germ cells, occurring after primordial germ cell differentiation in the early embryo, would be carried forward to the G2 generation without affecting G1 somatic cells. This is consistent with our observed pattern of disease in G1 and G2.

To conclude: Abundant ancestral nutrition appears to predict susceptibility to a range of cancers in men along a male-line pathway. Bias, chance or confounding from social and demographic factors did not explain these associations. The response in women is much more limited, visible only in a few instances, perhaps due to chance.

We therefore conclude that there is a possibility that an early molecular signal in men, reflecting pre-pubertal food abundance, is carried via sperm or seminal fluid to the next generation. Primordial germ cells in the new embryo (G1) are exposed to a genome-wide resetting of epigenetic marks, including sex-specific imprinting, in the first weeks of embryonal development^71,72^. Aberrant regulation of this process, for instance methylation of tumour suppressor genes or of DNA mismatch repair genes, may cause susceptibility to cancer in multiple organs in the subsequent G2 generation.

## Methods

### Ethics review

The linking of individuals to cancer registry and cause-of-death data was carried out by Statistics Sweden and all analyses were performed on data anonymized to researchers. The Regional Ethical Review Board of Stockholm reviewed and approved the study (dnr 2015/904-31/5; dnr 2016/933-32; dnr 2017/1043-32; dnr 2018/2273-32, dnr 2021-00726).

### Study population—the Uppsala Multigeneration study

All live births at the Academic Hospital in Uppsala 1915‒1929 constitute generation 1 (G1, born 1915‒1929). Their children who survived until the Census of 1960 could be followed-up and constitute generation 2 (G2, born 1932‒1990). The grandparental generation, denoted as generation 0 (G0, born 1851‒1914), was manually traced back from generation 1 by means of parish registers, hospital archives and the Swedish Death Index (6th edition compiled by genealogists and Statistics Sweden)^35,51^. We included only G2 and G1 men and women for whom there was information about two or more G0 ancestors, maternal or paternal. If two G1s had a child together, such a G2 had four grandparents (G0) in our data.

The tracing of G0 birth parishes was the basis for classifying G0 into type of childhood community (urban or rural). Information about regional harvest yields could be used to classify G0 by pre-pubertal food access. This was only possible for G0s born 1865‒1900. We, therefore, restricted our study of cancer events in G1 and G2 to those whose parents/grandparents were born 1865‒1900 (Table 1).

G0s growing up in the countryside were more dependent on annual crop outcomes than urban G0s. This is substantiated in the Supplementary Appendix to the paper in which we describe the historical context in which G0 grew up and cite the extensive economic-historical literature on this era in Sweden. It is also in line with findings from 19th century India^73^. Urban background is defined as growing up in one of the ten largest cities in Sweden, seven of which were sea ports. We therefore distinguish between rural backgrounds (both paternal or both maternal G0s grew up in the countryside) and urban (= urban + mixed) background.

The number of G2 with rural ancestry for whom there was full data on covariates in analyses of G2 cancer events was 8431 (4310 G2 men and 4121 G2 women). The equivalent number for those with urban/mixed ancestry was 3754 (1915 G2 men and 1839 G2 women). The number of G1 with rural ancestry for whom there was full data in analyses of G1 cancer events was 3781 (1960 men and 1821 women) (Table 1).

### Exposure—defined by regional harvest yields

Statistics Sweden published its annual review of harvest statistics every year from 1874 to 1910^74^. Harvest yields were classified on a scale from 0 to 10, where 0 is total crop failure and 10 is abundant harvest. This classification was applied to each of the 24 regions in Sweden. We defined two exposure categories as good/abundant (≥8.5) or poor/very poor (<5.0). Harvest yields between 5.0 and 8.5 constitute an intermediate category.

A G0 was considered as exposed to good/abundant food access if the regional harvest was 1) good/abundant any one year during his/her pre-pubertal period and 2) poor/very poor in no year. The exposure to poor/very poor food access is defined correspondingly. The terms ancestral food abundance or ancestral food shortage always refer to a G1/G2 whose G0 parent/grandparent had been thus exposed in pre-puberty. Around 90% of G1 and G2 were in an intermediate position, thus serving as a reference category (Supplementary Table 1). Further details in ref. 35.

Information about food access during G1 pre-puberty was only available through national harvest statistics, ranging from 1 (poor) to 5 (good). We generated a binary variable where *good access* was ≥3.3 any one year and *not good* was <3.3. G1 food access was only used as a control variable in analyses of the impact of G0 food access on G1 and G2 cancer events.

### Outcome—cancer events

Table 2 shows occurrence of cancer, where a cancer event is defined as a primary cancer in the Swedish Cancer Registry or a death from cancer registered as an underlying cause in the Cause-of-Death Registry. Thus, there were 3422 cancer events in G2 men and women, 2355 of which occurred among G2 with rural G0 ancestors. Among G1 there were 1599 cancer events in those with G0 parents growing up rurally (Table 1). As a sensitivity test, we instead included only a person’s first primary cancer registered in the Cancer Registry. Results in Table 2 remained, with only small changes in HRs. The hazard ratio for G2 men whose paternal grandfathers had good/abundant food access, changed from 3.05 (95% CI 1.98–4.69) to 2.60 (95% CI 1.70–4.00). See Supplementary Table 6.

Reclassification of all cancers registered in the Cancer Registry for the period 1961‒2017 into the 7th revision of the International Classification of Disease (ICD7) had been undertaken by the Swedish Cancer Registry. We were thus able to work with cancer outcomes classified as in ICD 7 for the entire period.

Problems in comparisons of ICD codes over time are noted in an internal Cancer Registry manual^46^. The long follow-up period highlights this problem. It is particularly difficult if one defines outcomes as three-digit ICD codes and less difficult when ICD codes are grouped into six broad groups, as below.

The traditional ICD7 classification of cancers suggests the following six broad groups, each of which we analysed as an outcome variable: malignant neoplasm of buccal cavity and pharynx (ICD7 codes 140–148); malignant neoplasm of digestive organs and peritoneum (150–159); malignant neoplasm of respiratory system (160–165); malignant neoplasms of breast and genito-urinary organs (170–181); malignant neoplasm of other and unspecified sites (190–199); neoplasms of lymphatic and hematopoietic tissues (200–209). If a person had a registration of cancer in two (or more) of these groups, each event was counted in analyses of its respective group. However, if a person had two or more cancer events within the same broad group of cancer, only the first event was counted as an event in that class. Total number of cancer events in Table 1 is therefore somewhat higher than the sum of grouped cancers in Tables 3 and 4.

Further specification of cancer diagnoses into three-digit ICD7 codes resulted in 51 cancer sites for men and 54 for women. Many of these had very few cancer events, making specific analyses less meaningful.

### Pre-pubertal susceptibility period

The existence of a susceptibility period just before puberty has been suggested in the previous studies^12,28,35,42,64^. Bygren et al. coined this period ‘the slow growth period’^64^. Following Bygren, we define the susceptibility period as 9‒12 years for boys and 8‒10 for girls and we denote it by pre-puberty. Individual variation and secular changes in the onset of puberty makes any specification of a pre-pubertal susceptibility period somewhat imprecise.

### Multiple comparisons—*testing one à priori hypothesis*

Our à priori focus, based on previous findings on mortality, was on the hypothesis that abundant access to food in paternal grandfathers would trigger a response in grandsons, but not in granddaughters. The null hypothesis was that there is no association whatsoever between ancestral (G0) food access and G2 cancer occurrence. We also tested for interaction with gender. We considered but chose not to adjust for multiple testing in Table 2.

We considered multiple testing in the following way. The likelihood of type 1 error (false positives=rejecting a true null hypothesis) would increase with the number of tests. An often-used approach to this problem is to change the level of significance (*α*), as in the Bonferroni correction, where *α/*number of tests is considered to be an appropriate level of significance. However, the likelihood of type 2 error (false negatives; accepting a false null hypothesis) would increase with a Bonferroni correction. Thus, a Bonferroni correction could be said to bias results towards the null, to be conservative.

Bonferroni correction for multiple testing is seen as more appropriate when there is no à priori hypothesis. We had no particular à priori hypothesis about whether any particular cancer group explained the findings in Table 2. Thus, we applied a Bonferroni corrected *p* value (0.05/24 = 0.0021) in analyses of the six broad groups of cancer in Table 3. These analyses suggested that two groups of male cancer were triggered as a specific response to ancestral food abundance, namely *cancers of the digestive systems and peritoneum* and *cancers in other and unspecified sites*. The elevated hazard ratio in the remaining four classes combined also survived a Bonferroni correction. Thus, chance is an unlikely explanation for these specific results.

Could the association between G0 food access and cancer in G2 men be based on a general susceptibility to cancer? The finding that HRs in six out of six group were >1 (*p* = 0.016) is compatible with a hypothesis of general susceptibility to cancer in men, linked to ancestral food abundance.

### Models

Hazard ratios were estimated by Cox proportional hazard models, with age as underlying time scale. Following a previous study^35^, confounding in analyses of G2 cancer occurrence was controlled for by adjusting for G2 birth year, sibship size, sibling order and loss of a parent before age 18, G1 social class in 1960, income and education in 1970, plus G0 birth year as a linear trend, with 95% CI based on sibling cluster robust standard errors. This set of factors is referred to as demographic and social factors in the tables.

G2 year of birth was grouped into five-year age bands as a categorical variable. Mother’s (maternal lineage) or father’s (paternal lineage) highest achieved education were collected from the Swedish Census 1970, grouped into elementary or more than elementary education; family income, obtained from the same source, grouped into quintiles based on a couple’s total earned income; social class, from the Swedish Census 1960 (non-manual workers, manual workers, farmers and entrepreneurs, and unknown); mother’s parity, which defined sibling position of G2 as 1, 2, 3–4, 5–6 or 7 and higher; sibship size; and finally, whether a parent died before the child was 18.

Models controlled for G0 birth years (as linear trends) and cluster standard errors at family level, to account for the fact that siblings and cousins share biological ancestors.

The food situation of the other paternal or maternal G0 grandparent and the G1 parent was also taken into account. In an additional analysis of G2 cancer occurrence we controlled for G0 men’s age at the birth of G1, to rule out confounding from G0 paternal age (Supplementary Table 5).

Confounding in analyses of G1 was controlled for by adjusting for G1 birth year and sibling order, G0 social class and marital status at G1 birth, G0 birth year as a linear trend, with 95% CI based on sibling cluster robust standard errors. Food access of the other G0 was also adjusted for. In analyses of G1 cancer occurrence, we controlled for G1 birth year (5-year groups), G1 family social class (six groups) and marital status at birth plus sibling position (defined as in G2 analyses).

To account for the different age distributions in G1 and G2 during follow-up we performed additional analyses of G1 by restricting G1 follow-up to the period 1961–1988. In this period mean age-at-risk for G1 was similar (65.5 years) to that in G2 analyses (Supplementary Table 4).

The final model, presented in tables, is based on individuals without missing data in covariate variables. All analyses were performed using R 4.1.1^75^.

### Reporting summary

Further information on research design is available in the Nature Portfolio Reporting Summary linked to this article.

## Supplementary information

Supplementary information

Reporting Summary

## Data Availability

Usage of social and cancer data is subject to restrictions imposed by the National Board of Health and Welfare and by Statistics Sweden, in accordance with Swedish and European legislation on privacy protection. Therefore, the data are not publicly available. Presently they can be accessed and analysed at a specified venue in Stockholm, at Stockholm University. Any request for data has to be approved by the Swedish Ethical Review Authority (registrator@etikprovning.se), following an application in Swedish language. An overriding principle is that the identity of individuals should not under any circumstances be revealed. Requests will be facilitated by the corresponding author and the coauthors (contact agneta.cederstrom@su.se). The Department of Public Health Sciences at Stockholm University will provide on-site office facility if data access is granted.
