# Supplementary material for: Food abundance in men before puberty predicts a range of cancers in grandsons
Source: Nat Commun. 2022 Dec 6;13:7507. doi: 10.1038/s41467-022-35217-1 (PMC9726939; doi:10.1038/s41467-022-35217-1)
Supplement: Supplementary file 1 — Supplementary information [file 41467_2022_35217_MOESM1_ESM.pdf]

Vågerö, D., Cederström, A. & van den Berg, G. Food abundance in men before puberty predicts a range of cancers in grandsons.

Supplementary Information. Cover page.

This document consists of

Supplementary Tables 1-6

Supplementary Appendix (about the historical context of harvesting in 19th century Sweden), including

Supplementary References

Supplementary Table 1. Proportions (%) of grandchildren (G2) and children (G1) exposed to ancestral good, intermediate or poor harvests based on rural plus urban samples with full covariate data.

Harvest yields<br><br>*G0 Ancestors*	**Good/abundant**	**Intermediate**	**Poor/very poor**
	**G2 men and women**		
**Paternal Grandfather**	4.9	89.5	5.6
**Paternal Grandmother**	4.7	91.4	3.9
**Maternal Grandfather**	4.7	89.8	5.5
**Maternal Grandmother**	4.9	88.1	6.2
	**G1 men and women**		
**Father**	4.8	89.6	5.6
**Mother**	4.6	90.6	4.8

Supplementary Table 2. Cancer occurrence among G2 men and women by G0 food access, restricted to G0s growing up in one of the ten largest cities or with mixed urban/rural background: hazard ratios (HR)* and 95% confidence intervals (95% CL) for total cancer using intermediate food access as reference group.

	G2 men			***G2 women***		
Food access of	**Hazard Ratio**	**95% CI**	**p-value**	**Hazard Ratio**	**95% CI**	**p-value**
**Paternal grandmother**						
**Good/ abundant**	1.07	0.36-3.12	0.907	1.13	0.47-2.73	0.790
**Poor/ very poor**	0.33	0.09-1.31	0.116	1.20	0.58-2.52	0.621
**Paternal grandfather**						
**Good/ abundant**	0.59	0.21-1.60	0.298	0.78	0.36-1.69	0.532
**Poor/ very poor**	0.15	0.02-1.01	0.052	1.02	0.56-1.85	0.943
Person years	*55 713*			*55 602*		
Cancer events	*222*			*304*		
	G2 men			***G2 women***		
	**HR**	**95% CI**	**p-value**	**HR**	**95% CI**	**p-value**
**Maternal grandmother**						
**Good/ abundant**	0.42	0.14-1.28	0.128	1.12	0.61-2.04	0.712
**Poor/ very poor**	0.97	0.42-2.26	0.940	1.24	0.69-2.23	0.467
**Maternal grandfather**						
**Good/ abundant**	0.71	0.36-1.42	0.330	0.88	0.52-1.49	0.635
**Poor/ very poor**	0.63	0.31-1.28	0.204	1.50	0.88-2.56	0.139
Person years	*52 173*			*51 654*		
Cancer events	*225*			*316*		

**controlling for social and demographic factors, plus G1 parent's food access.*

Supplementary table 3. Median age, mean age and standard deviation (sd) at the onset of cancer among all G2 and G1 men and women and among those of rural ancestry (= both G0 born rurally).

G2 men					
All G2 men			*G2 men of rural ancestry*		
**median**	**Mean**	**Sd**	**median**	**Mean**	**sd**
60	58.8	10.7	61	59.1	10.5
G1 men					
All G1 men			*G1 men of rural ancestry*		
**median**	**Mean**	**Sd**	**median**	**Mean**	**sd**
73	71.6	11.0	74	72.8	10.2
G2 women					
All G2 women			*G2 women of rural ancestry*		
**median**	**Mean**	**Sd**	**median**	**Mean**	**sd**
51	49.7	14.7	53	50.1	14.8
G1 women					
All G1 women			*G1 women of rural ancestry*		
**median**	**Mean**	**Sd**	**median**	**Mean**	**Sd**
71	68.4	14.4	72	69.8	14.3

Supplementary Table 4. ***Sensitivity analyses. Follow-up period of G1 restricted to 1961-1988.*** Cancer occurrence in G1 men by their G0 fathers´ food access in childhood, restricted to G0s with rural childhoods: hazard ratios* (HR), 95 % confidence interval (95% CI) and p-values for total cancer.

Food access of G0 father	***G1 men***			***G1 women***		
	**HR**	**95% CI**	**P-value**	**HR**	**95% CI**	**p-value**
**Good /abundant**	0.82	0.57-1.19	0.308	0.79	0.51-1.23	0.296
**Poor/very poor**	0.91	0.63-1.32	0.612	0.65	0.37-1.13	0.123

**controlling for social and demographic factors, plus G0 partner's food access.*

Supplementary table 5. *Sensitivity analysis of G2 paternal line analyses. Adding G0 age at G1 birth as control variable.* Cancer occurrence among G2 men and women by G0 food access, restricted to G0s with rural childhoods: hazard ratios (HR)* and 95% confidence interval (95% CI) for total cancer, using intermediate food access as reference group, during follow-up period of 1961–2017.

	G2 men			***G2 women***		
Food access of	**Hazard Ratio**	**95% CI**	**p-value**	**Hazard Ratio**	**95% CI**	**p-value**
**Paternal grandmother**						
**Good/ abundant**	1.07	0.54-2.10	0.848	0.97	0.64-1.47	0.876
**Poor/ very poor**	1.32	0.82-2.13	0.248	0.96	0.64-1.44	0.844
**Paternal grandfather**						
**Good/ abundant**	**3.04****	**1.97-4.70**	**5.03 E-07**	1.14	0.82-1.58	0.449
**Poor/ very poor**	0.82	0.48-1.38	0.463	1.21	0.42-1.74	0.301

**controlling for social and demographic factors, plus G0 partner's and G1 parent food access, plus G0 paternal age at G1 birth*
*** Estimate significantly >1*

Supplementary table 6. ***Sensitivity analysis. Based on G2's first primary tumour in the Cancer Registry.*** Cancer occurrence among G2 men and women by G0 food access, restricted to G0s with rural childhoods: hazard ratios (HR)* and 95% confidence limits (95% CL) for total cancer, using intermediate food access as reference group, during follow-up period of 1961-2017.

	G2 men			***G2 women***		
Food access of	**Hazard Ratio**	**95% CL**	**p-value**	**Hazard Ratio**	**95% CL**	**p-value**
**Paternal grandmother**						
**Good/ abundant**	1.01	0.54-1.91	0.843	1.04	0.65-1.69	0.859
**Poor/ very poor**	1.37	0.85-2.20	0.969	1.05	0.65-1.69	0.857
**Paternal grandfather**						
**Good/ abundant**	**2.60****	**1.70-4.00**	**1.3 E-6**	1.33	0.92-1.93	0.130
**Poor/ very poor**	0.87	0.49-1.83	0.625	1.29	0.87-1.91	0.210
**Maternal grandmother**						
**Good/ abundant**	0.75	0.22-2.50	0.702	1.10	0.75-1.62	0.623
**Poor/ very poor**	0.38	0.00-3.37	0.237	0.87	0.51-1.36	0.595
**Maternal grandfather**						
**Good/ abundant**	0.91	0.57-1.46	0.219	1.21	0.79-1.87	0.381
**Poor/ very poor**	1.27	0.85-2.02	0.387	1.14	0.64-1.96	0.605

**controlling for social and demographic factors, plus G0 partner's and G1 parent food access*

*** Estimate significantly >1.*

Supplementary appendix about the historical context of harvesting in 19th century Sweden

We describe the historical context of the rural society in which G0 individuals grew up. *We argue that conditions in rural areas were more dependent on crop outcomes, and, in particular, that in rural areas there was higher sensitivity to harvest volatility than in cities, because the rural areas were isolated and therefore had fewer opportunities to buffer (or insure against) shocks*. In the economic history literature, the seminal article by Burgess and Donaldson [1] compares areas in late 19th century India that are open for trade with the rest of the world with remote rural areas. They focus on the occurrence of harvest failures due to volatile weather and they find that open areas display less volatile real income and less volatile concurrent mortality than the remote rural areas. The volatility in the latter reduces substantially once they become connected to the railway network. In such cases the incidence of famines also decreases dramatically.

We now turn to rural areas in late 19th century Sweden. The fact that food shocks were not buffered is corroborated by the fact that Sweden witnessed a famine in 1868 which was triggered by unusually cold weather during a number of consecutive years and which was especially severe in rural and remote areas [2][3].

In rural areas, by far the most common job type was to be a so-called day laborer (unskilled farm worker doing manual work). Day laborers accounted for around 50% of the rural working class (see Lundh [4] for details). They were paid cash wages on a day-to-day basis. Benefits in kind (such as food) were not part of the earnings. Prado et al. [5] document volatility in wages of day laborers in Sweden over time. This evidence is based on county-level averages rather than individual data. However, it is striking that in the years most relevant to us (1880-1905), the coefficient of variation across counties fluctuated a lot. This is relevant for our purposes as volatility indicates that local transitory conditions have a real impact. The coefficient of variation displays a slightly decreasing trend from 0.18 to 0.16 but within the period 1880-1905 there are years where it is close to 0.25 and years where it is close to 0.10. In sum, the variation across counties did not vanish in this period, so there was no widespread convergence (which would have been difficult to reconcile with a high

relevance of harvest conditions). Also, there was no social safety net to buffer shocks and the dominant political view was that people should take care of their own misfortunes.

To augment this narrative, we look at the extent to which other mechanisms were in place that could mitigate the sensitivity to harvest shocks. In line with the literature, the two key potential mechanisms are (1) trade of goods (including food) and (2) migration. We first consider trade. Trade can serve to reduce differences in economic conditions between areas and reduce sensitivity to shocks within areas. We focus on Uppsala County as this is where most G0 individuals grew up. By analogy to Burgess and Donaldson [1] we examine the roll-out of railway infrastructure necessary for large-scale trade. Before railways were built, large-scale inland trade from rural areas across large distances was prohibitively expensive. Consequently, shortages of food could lead to nutritional deficits while the abundance of food in rural areas could lead to overconsumption of food. In 1870, the only train station in Uppsala County was in the city of Uppsala. In 1871, the station in the city of Enköping was built. After this, more tracks were built, but these were initially only designed for industry and mining purposes. Specifically, the Dannemora mines were crucial (see Andersson [6] for a comprehensive overview). In 1910 there were still only four railway lines in Uppsala County. These could only serve a limited number of villages. (A notable exception was the small village of Knutby which, in 1884, benefited from a railway line used for iron ore mining.) See Berger at al. [7] for more information.

Next, we consider migration. Residential moves in response to shocks may smoothen economic differences between areas, effectively making rural and urban areas more similar and mitigating the effects of harvest fluctuations. It is well known that Sweden faced mass migration starting in the late 1860s, until 1914. Most of this migration was external, i.e. out of Sweden. Karadja et al. [8] show that emigration took off in response to a sequence of cold years in the 1860s culminating in the 1868 famine. However, although Uppsala County had very low temperatures in the 1860s, it witnessed almost no emigration. Indeed, throughout the period 1868-1914, most emigration out of Sweden took place from cities rather than the countryside, and most of the emigration came from West and South Sweden. Karadja et al. [8] do not find strong evidence of selection in the composition of leavers versus remainers. In fact, there was virtually no emigration from Uppsala County (which lies in the East) and Enflo et al. [9] find almost no effects of emigration on wages in Uppsala County.

According to Enflo et al. [9], internal migration is mostly driven by unmarried adult men without children, which should be less relevant for our study design. It is unlikely that larger households (including those with sons in prepuberty) would migrate within Sweden in response to a single bad harvest in their region of origin or a single good harvest in the destination region. (Note that even if trade and migration were quantitatively relevant, our

findings would underestimate - in absolute value - the effects within rural communities of the absence of trade and migration, so our estimates of the causal effects of exposure would be conservative.)

This raises the question how unusually abundant yields were harvested. The harvest work was done by the day laborers. Many day laborers were employed during the summer half-year while they had other work in the same rural area during the winter half-year. Others were in fact permanently employed in agriculture for the whole year. Yet others were only employed in agriculture during peaks in agricultural production [4]. The day laborers' yearly starting and ending dates of employment in agriculture provided some leeway to adjust total labor supply to an increased demand. However, to obtain such leeway, the number of hours worked per day was more important. Hours could be adjusted easily and flexibly. With abundant harvests, day laborers could work more hours per day and their earnings would go up. With this they could buy more food at the market.

The relevance of the above narrative for our study design may be criticized if abundant harvests caused the day laborers to work so excessively hard that their health was negatively affected by that, either by exhaustion or by excess consumption of alcohol and cigarettes. However, this seems unlikely, and, moreover, we are exclusively interested in exposure before puberty. Young boys working as day laborers might have suffered from tiredness and/or some school absence in years of abundant harvests, but they would probably not have used the earnings for alcohol.

Another possible cause for concern may be that rural-urban differences reflect selective infant mortality. Lundh et al. [4] show that IMR were higher in urban areas than in rural areas in the late 19th century. This may imply that urban survivors were on average less frail. This might explain why results for our urban subsample are less pronounced than those for our rural subsample. However, this does not invalidate our study design, which focuses on the rural subsample.

Supplementary References

1. Burgess, R. and Donaldson, D., Can openness mitigate the effects of weather shocks? Evidence from India's famine era. *American Economic Review Papers and Proceedings*, 100:449–53 (2010).

2. Nelson, M. C., *Bitter bread: the famine in Norrbotten 1867-68.* Dissertation, Acta Universitatis Upsaliensis, Uppsala (1988).

3. Dolbhammer, G., Van den Berg, G.J. and Lumey, L.H., A re-analysis of the long-term effects on life expectancy of the Great Finnish Famine of 1866-68, *Population Studies* 67, 309–322 (2013).

4. Lundh, C. and Prado, S., Markets and politics: the Swedish urban–rural wage gap, 1865–1985, *European Review of Economic History*, Volume 19, Pages 67–87 (2015).

5. Prado, S., Lundh, C., Collin, K. and Enflo, K., Labour and the ‘law of one price’: regional wage convergence of farm workers in Sweden, 1757–1980, *Scandinavian Economic History Review*, 69:1, 41-62 (2021).

6. Andersson, O., *Järnvägar i Uppland, Varför blev de byggda och varför ligger de där de ligger? En översiktlig historik* (2001).

7. Berger, T. and Enflo, K., Locomotives of local growth: The short- and long-term impact of railroads in Sweden, *Journal of Urban Economics* 98, 124–138 (2017).

8. Karadja, M. and Prawitz, E., Exit, Voice, and Political Change: Evidence from Swedish Mass Migration to the United States, *Journal of Political Economy*, 127, no. 4 (2019).

9. Enflo, K., Lundh, C. and Prado, S., The role of migration in regional wage convergence: Evidence from Sweden 1860–1940. *Explorations in Economic History*, 52, 93–110 (2014).
