# Supplementary material for: Food abundance in men before puberty predicts a range of cancers in grandsons
Source: Nat Commun. 2022 Dec 6;13:7507. doi: 10.1038/s41467-022-35217-1 (PMC9726939; doi:10.1038/s41467-022-35217-1)
Supplement: Supplementary file 2 — Reporting Summary [file 41467_2022_35217_MOESM2_ESM.pdf]

nature portfolio

Corresponding author(s): Denny Vågerö

Last updated by author(s): Oct 28, 2022

Reporting Summary

Nature Portfolio wishes to improve the reproducibility of the work that we publish. This form provides structure for consistency and transparency in reporting. For further information on Nature Portfolio policies, see our Editorial Policies and the Editorial Policy Checklist.

Statistics

For all statistical analyses, confirm that the following items are present in the figure legend, table legend, main text, or Methods section.

n/a	Confirmed	
☐	☒	The exact sample size (*n*) for each experimental group/condition, given as a discrete number and unit of measurement
☒	☐	A statement on whether measurements were taken from distinct samples or whether the same sample was measured repeatedly
☐	☒	The statistical test(s) used AND whether they are one- or two-sided<br>*Only common tests should be described solely by name; describe more complex techniques in the Methods section.*
☐	☒	A description of all covariates tested
☐	☒	A description of any assumptions or corrections, such as tests of normality and adjustment for multiple comparisons
☐	☒	A full description of the statistical parameters including central tendency (e.g. means) or other basic estimates (e.g. regression coefficient) AND variation (e.g. standard deviation) or associated estimates of uncertainty (e.g. confidence intervals)
☐	☒	For null hypothesis testing, the test statistic (e.g. *F*, *t*, *r*) with confidence intervals, effect sizes, degrees of freedom and *P* value noted<br>*Give P values as exact values whenever suitable.*
☒	☐	For Bayesian analysis, information on the choice of priors and Markov chain Monte Carlo settings
☒	☐	For hierarchical and complex designs, identification of the appropriate level for tests and full reporting of outcomes
☐	☒	Estimates of effect sizes (e.g. Cohen's *d*, Pearson's *r*), indicating how they were calculated

Our web collection on statistics for biologists contains articles on many of the points above.

Software and code

Policy information about availability of computer code

Data collection	No software was used for data collection
Data analysis	For data analyses we used the program R 4.1.1

For manuscripts utilizing custom algorithms or software that are central to the research but not yet described in published literature, software must be made available to editors and reviewers. We strongly encourage code deposition in a community repository (e.g. GitHub). See the Nature Portfolio guidelines for submitting code & software for further information.

Data

Policy information about availability of data

All manuscripts must include a data availability statement. This statement should provide the following information, where applicable:

- Accession codes, unique identifiers, or web links for publicly available datasets
- A description of any restrictions on data availability
- For clinical datasets or third party data, please ensure that the statement adheres to our policy

Data availability statement: Usage of social and cancer data is subject to restrictions imposed by the National Board of Health and Welfare and by Statistics Sweden, in accordance with Swedish and European legislation on privacy protection. Therefore, the data are not publicly available. Presently they can be accessed and analysed at a specified venue in Stockholm, at Stockholm University. Any request for data has to be approved by the Swedish Ethical Review Authority (registrator@etikprovning.se) following an application in Swedish language. An overriding principle is that the identity of individuals should not under any

circumstances be revealed. Requests will be facilitated by the corresponding author and the coauthors (contact agneta.cederstrom@su.se). The Department of Public Health Sciences at Stockholm University will provide on-site office facility if data access is granted.

Human research participants

Policy information about studies involving human research participants and Sex and Gender in Research.

Reporting on sex and gender	We report results- cancer occurrence- separately for men and women
Population characteristics	We look for cancer occurrence 1961-2017 in men and women (born 1932-1990) who are the grandchildren of men and women born 1865-1900 and growing up in the Swedish countryside. Analyses look at whether or not paternal and maternal grandparents were exposed to food abundance or food shortage and relates this to cancer outcomes..
Recruitment	All children born at the Acade mic Hospital in Uppsala Sweden in 1915-1929 were recruited (generation 1). We traced their children and parents through civic registries, hospital archives and parish books.
Ethics oversight	Regional Ethical Review Board of Stockholm

Note that full information on the approval of the study protocol must also be provided in the manuscript.

Field-specific reporting

Please select the one below that is the best fit for your research. If you are not sure, read the appropriate sections before making your selection.

☒ Life sciences ☐ Behavioural & social sciences ☐ Ecological, evolutionary & environmental sciences

For a reference copy of the document with all sections, see nature.com/documents/nr-reporting-summary-flat.pdf

Life sciences study design

All studies must disclose on these points even when the disclosure is negative.

Sample size	The original population of individuals, generation 1, consists of everyone born and registered at the Academic Hospital in Uppsala Sweden in 1915-1929. All their children, generation 2, and their parents, generation 0, who could be traced were included in the study. Loss to follow-up in the cancer registry is considered very small.
Data exclusions	Individuals in generation 2, whose grandparents were born before 1865 or after 1900 were excluded , since publication of harvest statistics only cover the childhood of grandparents born 1865-1900. Covariate information was sometimes missing in analyses of confounding. Excluding such individuals did not change results.
Replication	Our study is registry based. We have not performed repeated experiments on animals.
Randomization	The availability of food in a specific region at a specific year could be seen, in part, as an experiment by nature. Variation in harvests and regions in Sweden during 1874-1910 contains a random element. Using harvest yields as "instrument variables" for food consumption exploits this random variation, thereby avoiding selection bias and reducing confounding.
Blinding	Researchers worked on anonymized data and had no information about the identity of the individuals who were followed up in the Swedish Cancer Registry and the Swedish Cause-of-Death Registry. The collection of data about independent variables and covariates was not influenced by any knowledge about future cancer events,

Reporting for specific materials, systems and methods

We require information from authors about some types of materials, experimental systems and methods used in many studies. Here, indicate whether each material, system or method listed is relevant to your study. If you are not sure if a list item applies to your research, read the appropriate section before selecting a response.

Materials & experimental systems

n/a	Involved in the study
☒	☐ Antibodies
☒	☐ Eukaryotic cell lines
☒	☐ Palaeontology and archaeology
☒	☐ Animals and other organisms
☒	☐ Clinical data
☒	☐ Dual use research of concern

Methods

n/a	Involved in the study
☒	☐ ChIP-seq
☒	☐ Flow cytometry
☒	☐ MRI-based neuroimaging
